# Referral to pulmonary rehabilitation and palliative care services in people with idiopathic pulmonary fibrosis in England, 2010–2019

**DOI:** 10.1038/s41533-024-00387-6

**Published:** 2024-10-09

**Authors:** Ann D. Morgan, Hakeem Khan, Peter M. George, Jennifer K. Quint

**Affiliations:** 1 School of Public Health, Imperial College London, London, UK; 2 Interstitial Lung Disease Unit, Royal Brompton Hospital and Harefield NHS Foundation Trust, London, UK; 3 National Heart and Lung Institute, Imperial College London, London, UK

**Keywords:** Rehabilitation, Palliative care

## Abstract

The benefits of pulmonary rehabilitation (PR) and palliative care (PC) as non-pharmacological therapies for people with idiopathic pulmonary fibrosis (IPF) are increasingly being recognised but in the UK the proportion of people with this life-limiting condition who are referred to such services is thought to be low. This retrospective cohort study aimed to describe trends in referrals to PR and PC services among people with IPF over a 10-year period and to identify factors associated with non-referral. Our study cohort was drawn from the UK’s pseudonymised Clinical Practice Research Datalink (CPRD) Aurum primary care database and comprised 17,071 individuals diagnosed with IPF between 2010 and 2019. While 12.0% of IPF patients were offered a referral to PR, less than 2% completed a PR programme. Around a fifth (19.4%) received a referral to generic PC support services; however, this is well below reported PC referral rates for lung cancer patients. Moreover, the majority of PC referrals occurred late; among those who died, 31% were referred within a month and 70% within 6 months of death. Referrals to PR and PC had however increased (by around 2–fold and 4-fold, respectively) over the course of the study period. Factors associated with non-referral to PR included female sex, older age and co-diagnosis of dementia; barriers to PC referral included being female or of Asian or Black ethnicity. We also found evidence of regional differences in referrals. These findings confirm that PR and PC service provision for people with IPF across England is suboptimal.

## Introduction

Idiopathic pulmonary fibrosis (IPF) is a relatively rare chronic condition characterised by scarring of the lung parenchyma resulting in a gradual, irreversible decline in lung capacity and ability of the lungs to oxygenate blood. The most common manifestations are increasing breathlessness and a persistent cough^1,2^. Once diagnosed, the prognosis is typically poor, with an average survival time without treatment in the UK of just 3–5 years^3^. While IPF is rare in people under the age of 45 years, incidence increases with age^1,3^.

For a variety of reasons, it has been difficult to reliably characterise the burden of IPF, and published estimates of incidence and prevalence both in the UK and elsewhere are highly variable^3–5^. Analysis of UK electronic health records for the period 2008–2018 suggest that IPF incidence most likely lies between 15 and 16 cases per 100,000 person-years, which scaled up to the UK population implies that around 8000–9000 new cases of IPF are diagnosed each year^6^. Several studies have indicated that incidence and prevalence of IPF have increased over the past two decades, although the precise reasons for the observed trends remain largely speculative at this time^3,6,7^.

A growing body of qualitative research supports a holistic approach to the management of people living with a diagnosis of IPF^8^. Both pulmonary rehabilitation (PR) and palliative care (PC) are now widely perceived important components of such an approach, one which delivers an integrated package of care for both patients and their carers^9,10^. Although PR was originally devised to support individuals with chronic obstructive pulmonary disease (COPD), in recent years evidence of benefits in the IPF population has begun to emerge^11^. Likewise, access to integrated PC services – which encompass not just symptom management but also address the emotional, social and spiritual needs of people with terminal illnesses – has been shown in several studies to improve the quality of life of people with IPF^12,13^. Currently, PC tends to be introduced late, often within the last 30 days of life and during an episode of acute respiratory failure, with the result that a high proportion of people with IPF die in hospital settings^14,15^. This has led many to advocate for earlier integration of PC into standard care for IPF patients^16^.

Despite growing evidence of benefit, especially in terms of patient-centred outcomes and quality of life, PR and PC service provision is believed to be poor among people with IPF, with referral rates typically much lower than those for other respiratory conditions such as lung cancer and COPD^8,17^. National registry data from several countries, including Germany, Spain and the USA suggest that less than 20% of people with a diagnosis of IPF are offered a PC referral and less than 10% attend PR^8^.

Reliable data on referrals to PR and PC among the wider IPF patient population are currently lacking. The primary aim of this study was therefore to describe UK trends in referral to PR programmes and PC services among people with IPF using routinely-collected health data and to provide baseline (pre-pandemic) data on service use to serve as a benchmark for monitoring improvements in provision. A secondary objective was to investigate associations between demographic and patient factors and PR and PC referrals. By identifying potential barriers to referral, it is hoped that this study will help to increase access to PR and PC services in people with IPF.

## Results

### Cohort characteristics

A total of 17,071 people met our inclusion criteria and were included in the study cohort (Supplementary Fig. S1). The majority of the cohort was male (62.6%) and the median age at IPF diagnosis was 76.7 years. The overwhelming majority of study participants had a history of smoking (88.5%) and the most common comorbidities were IHD (27.2%), depression/anxiety (25.7%), diabetes (23.7%) and GORD (23.2%). Table 1 summarises the key characteristics of the study population as a whole, and with respect to post-IPF diagnosis referral to PR and PC services (separately).

**Table 1:** Demographic and clinical characteristics of study participants stratified by referral status.

Characteristic	Whole cohort n (%)	Pulmonary rehabilitation		Palliative care	
		Not referred n (%)	Referred n (%)	Not referred n (%)	Referred n (%)
Number of patients	17,071	15,029	2042	13,756	3315
*Demographic and lifestyle*					
Sex					
Female	6383 (37.4%)	5734 (38.2%)	649 (31.8%)	5227 (38.0%)	1156 (34.9%)
Male	10,688 (62.6%)	9295 (61.9%)	1393 (68.2%)	8529 (62.0%)	2159 (65.1%)
Age at IPF diagnosis (years)					
Median (IQR)	76.7 (69.6–82.7)	77.1 (69.9–83.1)	74.1 (67.1–79.4)	76.5 (69.1–82.5)	77.6 (71.4–83.4)
Age group					
40–59	1260 (7.4%)	1082 (7.2%)	178 (8.7%)	1122 (8.2%)	138 (4.2%)
60–69	3204 (18.8%)	2696 (17.9%)	508 (24.9%)	2638 (19.2%)	566 (17.1%)
70–79	6502 (38.1%)	5611 (37.3%)	891 (43.6%)	5194 (37.8%)	1308 (39.5%)
≥80	6105 (35.8%)	5640 (37.5%)	465 (22.8%)	4802 (34.9%)	1303 (39.3%)
BMI					
Underweight (<18.5)	465 (2.7%)	412 (2.7%)	53 (2.6%)	349 (2.5%)	116 (3.5%)
Normal (18.5–24.9)	3765 (22.1%)	3302 (22.0%)	463 (22.7%)	2955 (21.5%)	810 (24.4%)
Overweight (25.0–29.9)	4498 (26.3%)	3866 (25.7%)	632 (31.0%)	3626 (26.4%)	872 (26.3%)
Obese (≥30.0)	3389 (19.9%)	2868 (19.1%)	521 (25.5%)	2816 (20.5%)	573 (17.3%)
Missing	4954 (29.0%)	4581 (30.5%)	373 (18.3%)	4010 (29.2%)	944 (28.5%)
Smoking status					
Ex-smoker	12,409 (72.7%)	10,954 (72.9%)	1455 (71.3)	9957 (72.4%)	2452 (74.0%)
Current smoker	2704 (15.8%)	2237 (14.9%)	467 (22.9%)	2194 (16.0%)	510 (15.4%)
Never smoker	1946 (11.4%)	1826 (12.2%)	120 (5.9%)	1595 (11.6%)	351 (10.6%)
Missing	12 (0.1%)	12 (0.08%)	0 (0.0%)	10 (0.07%)	2 (0.06%)
Ethnicity					
White	15,782 (92.3%)	13,866 (92.4)	1916 (93.8%)	12,663 (92.1%)	3119 (94.1%)
Asian	847 (5.0%)	762 (5.1%)	85 (4.2%)	714 (5.2%)	133 (4.0%)
Black	185 (1.1%)	169 (1.1%)	16 (0.8%)	167 (1.2%)	18 (0.54%)
Mixed	58 (0.3%)	50 (0.3%)	8 (0.4%)	49 (0.4%)	9 (0.27%)
Other	91 (0.5%)	79 (0.5%)	12 (0.6%)	80 (0.6%)	11 (0.33%)
Missing/Unknown	108 (0.6%)	103 (0.7%)	5 (0.25)	83 (0.60%)	25 (0.75%)
Region					
North East	735 (4.3%)	631 (4.2%)	104 (5.1%)	588 (4.3%)	147 (4.4%)
North West	4338 (25.4%)	3685 (24.5%)	653 (32.0%)	3459 (25.2%)	879 (26.5%)
Yorkshire/Humber	736 (4.3%)	646 (4.3%)	90 (4.4%)	606 (4.4%)	130 (3.9%)
East Midlands	303 (1.8%)	260 (1.7%)	43 (2.1%)	242 (1.8%)	61 (1.8%)
West Midlands	2924 (17.1%)	2623 (17.5%)	301 (14.7%)	2321 (16.9%)	603 (18.2%)
East of England	564 (3.3%)	500 (3.3%)	64 (3.1%)	421 (3.1%)	143 (4.3%)
London	1863 (10.9%)	1673 (11.1%)	190 (9.3%)	1553 (11.1%)	340 (10.3%)
South East	3354 (19.6%)	2982 (19.8%)	372 (18.2%)	2727 (19.8%)	627 (18.9%)
South West	2016 (11.8%)	1815 (12.1%)	201 (9.8%)	1676 (12.2%)	340 (10.5%)
Missing	238 (1.45)	214 (1.4%)	24 (1.2%)	193 (1.4%)	45 (1.4%)
IMD (quintiles)					
Least deprived	3521 (20.6%)	3150 (21.0%)	371 (18.2%)	2847 (20.7%)	674 (20.3%)
Low deprivation	3670 (21.5%)	3244 (21.6%)	426 (20.9%)	2960 (21.5%)	710 (21.4%)
Moderate deprivation	3199 (18.8%)	2832 (18.8%)	367 (18.0%)	2550 (18.5%)	649 (19.6%)
High deprivation	3207 (18.8%)	2816 (18.7%)	391 (19.2%)	2567 (18.7%)	540 (19.3%)
Most deprived	3454 (20.3%)	2969 (19.8%)	4485 (23.8%)	2815 (20.5%)	639 (19.3%)
Missing	20 (0.1%)	18 (0.12%)	2 (0.1%)	17 (0.12%)	3 (0.09%)
*Comorbidities*					
COPD	2774 (16.5%)	1951 (13.0%)	823 (40.3%)	2138 (15.5%)	636 (19.2%)
Asthma (adult onset)	2903 (17.1%)	2442 (16.2%)	461 (22.6%)	2354 (17.1%)	549 (16.6%)
Lung cancer	137 (0.8%)	114 (0.76%)	23 (1.1%)	90 (0.65%)	47 (1.4%)
PAH	313 (1.8%)	279 (1.9%)	34 (1.7%)	237 (1.7%)	76 (2.3%)
GORD	3954 (23.2%)	3442 (22.9%)	512 (25.1%)	3176 (23.1%)	778 (23.5%)
Hernia	2439 (14.3%)	2141 (14.3%)	298 (14.6%)	1926 (14.0%)	513 (15.5%)
Heart failure	1842 (10.8%)	1661 (11.1%)	181 (8.9%)	1430 (10.4%)	412 (12.4%)
IHD	4649 (27.2%)	4131 (27.5%)	518 (25.4%)	3613 (26.3%)	1036 (31.3%)
Diabetes	4045 (23.7%)	3442 (22.9%)	473 (23.2%)	3237 (23.5%)	808 (24.4%)
Stroke	1602 (10.7%)	1602 (10.7%)	211 (10.3%)	1441 (10.5%)	272 (11.2%)
Dementia	445 (2.7%)	436 (2.9%)	19 (0.9%)	358 (2.6%)	97 (2.9%)
Depression/anxiety	4383 (25.7)	3806 (25.3%)	582 (28.5%)	3522 (25.6%)	866 (26.1%)

*BMI* body mass index, *COPD* chronic obstructive pulmonary disease, *GORD* gastro-oesophageal reflux disease, *IHD* ischaemic heart disease, *IMD* index of multiple deprivation, *PAH* pulmonary artery hypertension

### Pulmonary rehabilitation

#### Referral patterns

During follow up, a total of 2042 people (12.0% of the cohort) were referred to PR following an IPF diagnosis. More men than women were referred to PR, not only in terms of absolute numbers (1393 men were referred versus 649 women) but also in terms of the proportion referred (13.0% of men were referred to PR whereas just 10.2% of women had a PR referral). Nearly half of all referrals (43.6%) occurred in the 70–79 age group (n = 891) and around a fifth (22.3%) of those referred were current smokers. Comorbidities such as COPD, asthma and depression/anxiety were more common in the referral group (PR group) than in the non-referral group (non-PR group). For instance, 40.9% of the PR group (836 of 2042) also had COPD but only 12.9% of the non-PR group also had COPD. Dementia was less common in the PR group than in the non-PR group (Table 1).

**Fig. 1:**
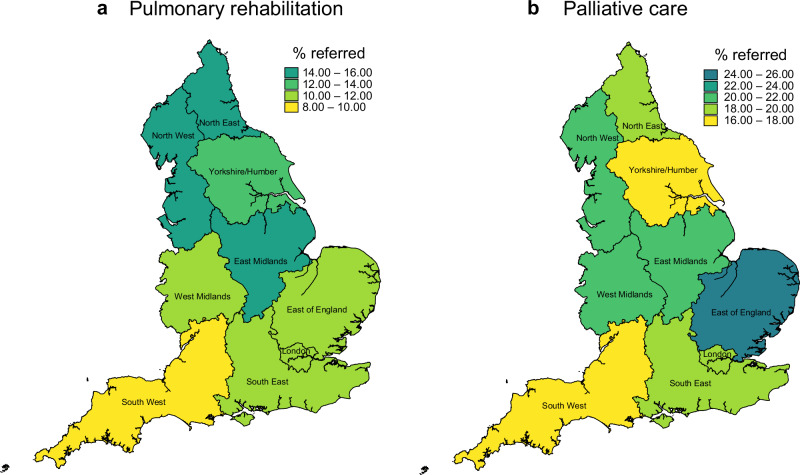
Referrals by region, 2010-2019. Percentage of patients with a diagnosis of IPF referred to **a** pulmonary rehabilitation and **b** palliative care servcies.

Regional variations in PR referrals were in evidence. The North West region was responsible for referring the greatest number of people, followed by the South East region (n = 653 and n = 372, respectively). In terms of the proportion of IPF cases with a referral, the North West again ranked highest, achieving a referral proportion of 15.1% for the study period overall which compares with an average of 12.0% for England (Fig. 1a; Supplementary Table S1). The South West recorded the lowest proportion of PR referrals. London also had a referral proportion that was below the average for England (10.2%).

**Fig. 2:**
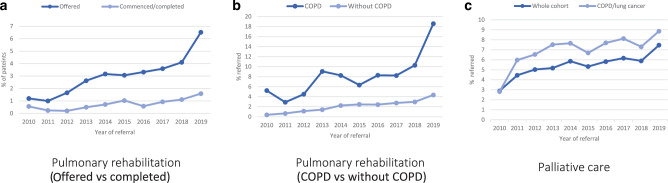
Temporal trends in referrals, 2010-2019. Number of patients referred to pulmonary rehabilitation (**a**, **b**) and pulmonary care services (**c**) in a given year expressed as a percentage of those with a diagnosis of IPF in the same year.

Temporal analyses suggested that the proportion of IPF patients who were referred to PR increased over the period of our study (Fig. 2a). Only 15 of 1246 people diagnosed with IPF in 2010 (1.2%) were offered PR. In 2014, 181 out of 5718 patients or 3.2% were referred (this includes referrals among those diagnosed in 2014 and those previously diagnosed and under follow up at the start of 2014); by 2019 this proportion had risen to 6.5% (574 out of 8811 people diagnosed in 2019 plus people previously diagnosed and under follow up at the start of 2019). When we restricted our analysis to those who had commenced or completed PR following an IPF diagnosis (as opposed to offered a referral), the proportion was considerably lower, under 1% in 2010 rising to just 1.6% by 2019 (Fig. 2a). Referral proportions were higher in those who also had been previously diagnosed with COPD, but even among those without a COPD diagnosis, we observed a steady increase in the referral proportion over time (Fig. 2b).

#### Factors associated with referral

**Fig. 3:**
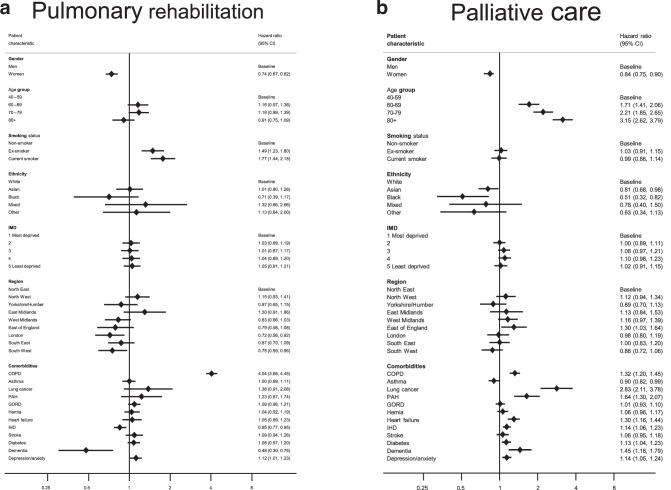
Hazard ratios for referral. Adjusted hazard ratios(HRs)^a^ for referral to **a** pulmonary rehabilitation and **b** palliative care services in a cohort of people diagnosed with IPF between 2010 and 2019. BMI body mass index, CI confidence interval, COPD chronic obstructive pulmonary disease, GORD gastro-oesophageal reflux disease, HR hazard ratio, IHD ischaemic heart disease, IMD index of multiple deprivation, PAH pulmonary artery hypertension. ^a^Mutually adjusted for all other listed variables with the exception of body mass index.

In a univariable analysis, younger age, male sex, smoking history, region, and comorbid COPD, asthma, GORD, smoking history, region, and comorbid COPD, asthma, GORD or depression/anxiety were among the factors that were positively associated with a referral to PR (Supplementary Table S2). Factors which remained strongly positively associated with PR referral after adjustment for potential confounders were male sex, smoking history and a prior diagnosis of COPD (Fig. 3a; Table S2). co-existing dementia remained strongly associated with non-referral (HR_adj_= 0.48, 95%CI = 0.30–0.76), while older age (over 80 years) and presence IHD were also identified as potential contributory factors to non-referral. Regional disparities also remained evident in the adjusted analysis, with the regions in the south of the country tending to do less well than those in the north. In subgroup analyses, in which we estimated HRs for PR referral for people with and without a prior diagnosis of COPD, female sex and co-diagnoses of dementia and IHD remained associated with non-referral irrespective of COPD status; some variation in the strength and direction of regional associations between the two subgroups was observed, adding to the evidence for regional disparities in PR referral practices (Supplementary Table S3).

### Palliative care

#### Referral patterns

Nearly a fifth of the cohort (19.4%) was referred to PC support services (2159 men and 1156 women). Nearly 80% of all PC referrals occurred in the over-70s (Table 1). Lung cancer and pre-existing cardiovascular conditions ranked among the comorbidities that were more prevalent in the PC group relative to the non-PC group. Among the small proportion who were diagnosed with lung cancer (n = 137, 0.8%), around a third (n = 47, 34%) received a referral to PC support. Among those who did not have a co-diagnosis of lung cancer (n = 16,934), less than a fifth were offered PC (n = 3268 or 19.3%).

In terms of the proportion of people with a GP-recorded IPF diagnosis who received a referral to PC support services, the East of England was the best performer (25.4%). The South West and the Yorkshire/Humber regions were below the average for England, with referral proportions of 16.9% and 17.7%, respectively (Fig. 1b; Table S1). The proportion of IPF patients with a PC referral increased from 2.9% in 2010 to 5.9% in 2014, peaking at 7.5% in 2019 (Fig. 2c). While the direction of temporal trends in PC referrals was similar for men and women, women lagged behind men in terms of referral to both PR and PC throughout the study period **(**Supplementary Fig. S2a, b).

#### Factors associated with referral

Univariate Cox models identified female sex, Asian and Black ethnicity and higher BMI (>25) as potential risk factors for non-referral to PC support services. Conversely, older age, underweight and the presence of comorbidities, in particular lung cancer and cardiovascular diseases, were identified as factors that favoured a PC referral (Fig. 3b; Table S2). Most of these risk factors remained significant in the fully-adjusted analysis; among the factors most strongly associated with non-referral were female sex (HR_adj_= 0.84, 95%CI 0.78–0.90) and Black ethnicity (HR_adj_= 0.51, 95%CI 0.32–0.82) while older age, and co-existing lung cancer, dementia and cardiovascular comorbidities remained associated with PC referral. We did not find strong evidence of geographical or SES-related disparities in referrals for PC support after accounting for confounding. Among the factors favouring referral, a co-diagnosis of lung cancer emerged as the strongest (HR_adj_= 2.83, 95%CI, 2.11–3.78); however, repeating the Cox analysis separately for people with and without lung cancer proved inconclusive owing to the low number of people with a prior lung cancer diagnosis in our study cohort (Supplementary Table S4).

#### Timing of PC referrals relative to death

Just under half of our study cohort (n = 7918) died before the end of our study period, of whom 2658 (33.6%) received a referral to PC services. Of these 2658 patients, 515 (19.4%) also had COPD and 38 (1.4%) also had lung cancer. Among this group, the median time lapse between PC referral and death was 73 days (IQR, 20–229 days). Moreover, among the 2658 recipients of PC services, around 70% were referred within 6 months of their death; only 15% had a referral more than a year before they died.

**Fig. 4:**
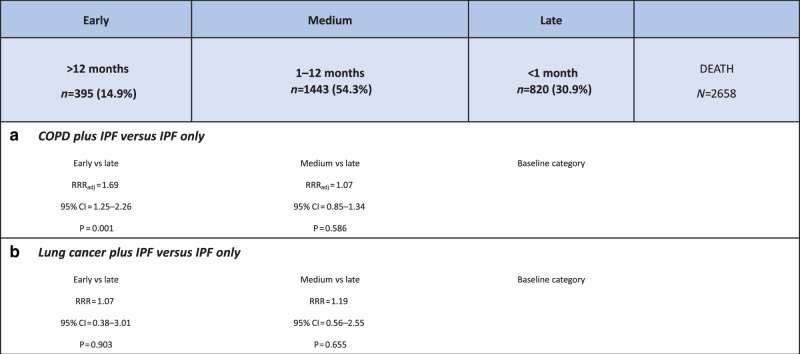
Timing of palliative care referrals relative to death. Relative risk ratios (RRRs) for early referral comparing **a** patients with and without comorbid COPD and **b** patients with and without comorbid lung cancer. COPD chronic obstructive pulmonary disease, RRR relative risk ratio.

Results of the multinomial logistic regression analysis, in which late PC referral (<1 month prior to death) was selected as the baseline category, are shown in Fig. 4. We found that patients who also had a COPD diagnosis were more likely to receive an “early” PC referral (at least a year pre-death) than those who did not also have COPD (RRR = 1.69, 95%CI,1.27–2.26). While the relative risk ratio (RRR) of an early PC referral (relative to a late referral) comparing those with and without lung cancer was greater than 1 (Fig. 4), suggesting that people with lung cancer were also more likely to be referred early than those without, our results failed to reach statistical significance. Older age was the only other factor (≥70 years) that was likely to result in an earlier referral to PC support; there was no evidence to suggest that sex, SES, region or ethnicity influenced early versus late referrals in this patient cohort.

#### Sensitivity analyses

Neither addition of a cluster option to account for variability between Aurum practices nor adjustment for year of IPF diagnosis materially altered our main findings. While we observed small changes in the magnitude and statistical significance of the HRs for several covariates in our multivariate models, most noticeably for some of the more marginal associations, those factors which emerged as being strongly associated retained statistical significance across the full range of our sensitivity analyses.

## Discussion

This study provides confirmation of a widely-held perception that among the IPF patient population, PR and PC referral rates are extremely low. Out of a study population comprising 17,071 people registered at GP practices across England and diagnosed with IPF during the 10-year period 2010–2019, only 12% were considered for and/or offered a PR referral during follow up. The proportion whose primary records indicated that they had commenced or completed PR was lower still, just 1–2%. The percentage receiving a referral to PC support services was higher, but at around 20% is significantly below the 75% reported for cancer patients. Moreover, the majority of PC referrals were late; among those who died, 31% were referred only within a month of death, and 70% within 6 months. On the upside, our study revealed that referrals to PR and especially to PC have increased over the 10 years to 2019. We also identified several factors that were associated with non-referral; for PR the most significant factors were female sex, older age and co-diagnosis of dementia. In the case of PC, the most significant barriers to referral were being female or being of Asian or Black ethnicity.

Published data on referrals to PC in people with IPF and other fibrosing ILDs are sparse but our rates are in line with those reported in other countries. Our overall estimate of 21% for PC referrals lies within the range reported by a systematic review of 10 studies (0% to 38%)^18^, and compares favourably with US data which suggest PC referral rates of around 14%^19,20^. The US studies, while based on data from a single centre, are noteworthy in the context of our study in that they also found that a high proportion of PC referrals occurred late (70% within 30 days of death)^19^ and that PC referral was associated with a higher proportion of hospice and home deaths^20^.

Our estimate of 12% for PR referrals lies towards the low end of the range reported in the literature. Most reported estimates stem from registry-based cohorts; analysis of US IPF-PRO registry data for example suggested a PR referral rate of 19.5%^21^. Our estimate is considerably lower than that published in the recent 10-year review of UK BTS ILD registry data which indicated that 89% of all IPF cases included in the registry between 2013 and 2022 had their PR needs assessed and of those, 57% were referred to PR^22^. There are several reasons why PR referral rates based on primary care data might be lower than those derived from registry data, at least in the UK context where IPF is largely managed in secondary care and specialist centres. While we acknowledge that our referral proportion of 12% might well be an underestimate (due to under-recording in primary care), we would argue that our estimate is a truer reflection of the historically low PR provision among the UK IPF patient population than that suggested by registry data. Several researchers have attributed low provision to past inconsistencies in the evidence base regarding the clinical benefits of PR in people with IPF, which led to discrepancies in older national clinical guidelines with some UK bodies, notably the National Institute for Health and Care Excellence (NICE)^23^ recommending PR more widely than others^24^. Interestingly, both our analysis and that of BTS registry data revealed increases of a similar magnitude in the proportion of patients referred to PR over time.

Age and sex were found to influence the likelihood of referral to both PR and PC. Not surprisingly, patients in the oldest age category, 80+ years, were more than three times more likely to be referred to PC services than people diagnosed with IPF in their forties and fifties (HR_adj_= 3.15, 95%CI 2.62–3.79). In contrast, people in their eighties were less likely to be referred to PR than their younger counterparts. This too is not altogether surprising given the complexity of older patients’ health profiles and aligns with studies conducted in the COPD population^25^. The male-female disparity in PR referral also mirrors findings in the COPD population^25^. What is perhaps more surprising is the male excess in PC referral given that some studies from the cancer literature have suggested that women are more likely to choose palliative care than men^26^. That said, the issue of gender differences in PC are likely multifaceted and possibly disease-specific and remain poorly understood.

In terms of demographic and socioeconomic factors, we observed some regional variations in referral to both PR and PC, with regions outside of London often performing marginally better, including for example the North West and North East (PR) and the East of England (PC). We also found that people from ethnic minorities were less likely to receive a PC referral, but we found no evidence to suggest that socioeconomic factors were associated with either PR or PC referrals. While a decreased PC referral rate in ethnic minorities is supported by the literature relating to other chronic respiratory conditions as well as palliative care more generally^27,28^, our lack of an association with IMD is contrary to the COPD literature which indicates that the most deprived are more likely to receive PR referrals, while the least deprived are more likely to receive PC referrals^29,30^. Unravelling the systemic and individual patient factors which underpin inequalities in PR and PC referral is not an easy task but warrants further investigation. Based on the findings of our analysis, we posit that factors other than socioeconomic status may be contributing to regional differences in referrals to PR and PC services by people with IPF, such as low awareness among healthcare providers of benefit, under-resourced and inefficient services which fail to address the specific needs of IPF patients, and lack of accessibility of specialist services.

In terms of the factors which increased the likelihood of a referral, the presence of selected comorbidities, including COPD, lung cancer and cardiovascular diseases emerged as strong determinants. In the case of PR, a prior diagnosis of COPD increased the likelihood of a PR referral four-fold, and in the group without COPD, the referral proportion for our study period overall was just 8.5%. Given that PR programmes were originally designed with the needs of the COPD patient population in mind, such a strong positive association between COPD and PR referral in an IPF population is to be expected. Moreover, as people with IPF are often misdiagnosed with COPD prior to receiving their IPF diagnosis, it is likely – because of our study design–that we may have underestimated the number of PR referrals in people who have been diagnosed with both conditions, omitting those referrals which were made prior to IPF diagnosis.

A co-diagnosis of COPD was also identified as a factor which favoured a PC referral (HR_adj_= 1.32; 95%CI: 1.20–1.45). The presence of cardiovascular diseases, in particular pulmonary hypertension and heart failure, as well as dementia, were also significantly associated with PC referrals after adjustment for age, sex and other confounders. However, lung cancer stood out as having the strongest association, nearly trebling the likelihood of a PC referral (HR_adj_=2.83, 95% CI: 2.11–3.78). This begs the question whether PC support in IPF is driven more by the co-existence of malignant disease for which palliative care programmes are better established. This question has also arisen in the context of COPD, and a study published in 2018 which examined trends in PC referrals in a COPD cohort over the period 2008–2014 concluded that the vast majority of patients were not provided with PC support services in their last year of life; when it was, it appeared to be related to their co-diagnosis of lung cancer rather than to the severity of their airways disease^31^. COPD patients who also had lung cancer were also more likely to receive PC support in a timely manner^31^. Our study provides some evidence that similar factors are playing out in IPF. However, because of the low numbers of patients with a dual diagnosis we were unable to establish whether a lung cancer diagnosis was more likely to result in an earlier (<1year) – as opposed to a late (<1 month) – referral to PC support. We did find that co-existing COPD favoured earlier PC referral over late, which might be a reflection of recent improvements in PC referral rates in people with non-malignant life-limiting chronic lung disease.

### Study limitations

Our ability to reliably identify referrals to pulmonary rehabilitation and palliative support services in routinely-collected primary care data is the main weakness of this study. This ultimately relies on the quality of coding of patient consultations by GPs and practice staff. However, since referral to PC services is one of the NICE Quality and Outcomes Framework (QOF) indicators (which means that GPs are incentivised to record PC referrals) we have greater confidence in the quality of the recording of referrals to generalised PC support services. However, we did not consider individual components of PC support, including those which may be of particular relevance to IPF patients such as prescription of medications to palliate breathlessness. In addition, since this study is based in primary care records only, we were not able to directly capture referrals to PR and PC that were made in secondary care. While we acknowledge this as a potential weakness of our study, we believe that a high proportion of referrals made in outpatient clinics and during hospital admissions will also have been recorded in patients’ GP records. Possibly the weakest capture is the number of completed PR programmes, but even allowing for this, our study suggests that an extremely low proportion of people with IPF are starting and completing PR.

While we are confident that we have identified key drivers of referral to PR and PC services in IPF, there are inevitably some potential factors that we have not been able to explore due to lack of data (or high levels of missingness), for example, measures of disease severity (breathlessness, oxygen supplementation) and eligibility for antifibrotic therapy. We also accept that our use of IMD quintiles as our measure of SES is potentially flawed, given that this is a broad measure which aggregates several aspects of deprivation. Nor can we exclude the possibility of residual confounding. We used GP practice as a random effect in our Cox models to account for the clustering of patients within practices but accept that there may also be clustering at the individual GP level. However, it seems a reasonable assumption that in the context of PR and PC services, referral decisions are more likely to vary between practices than between individual GPs. Finally, we note that our geographical focus on England necessitates caution when extrapolating the findings to other UK nations.

In sum, this analysis revealed that despite improvement since 2010, referrals to PR programmes and PC services among people with IPF in 2019 remain low, well below those for other chronic life-limiting lung conditions. The gender gap in service provision, with women less likely than men to receive a referral to PR and PC is a key concern and warrants further investigation. While for this patient population the benefits of PR and early referral to PC services are increasingly being recognised and embodied in current guidelines, it is evident that there needs to be substantial investment in service provision if the currently unmet demand for comprehensive and patient-centred PR and PC services is to be met.

## Methods

### Data sources

This study used the Clinical Practice Research Datalink (CPRD) Aurum database (February 2022 build), a curated set of patient-level electronic health records provided by 1,345 participating GP practices, which collectively deliver primary health care to one fifth (19.9%) of the UK population. CPRD Aurum provides pseudonymised, longitudinal SNOMEDCT-coded information on individuals’ demographic characteristics (e.g., sex, age), lifestyle behaviours (e.g., BMI, smoking status), symptoms and clinical diagnoses, medication and vaccination histories, as well as results of laboratory tests and details of referrals to secondary care^32^. The CPRD Aurum dataset has been shown to be representative of the UK population in terms of age, sex and ethnicity^32^. CPRD Aurum data are routinely linked to other health-related data sets, including the Index of Multiple Deprivation (IMD), a post-code level measure of social deprivation. Such linkages are currently limited to 80% of Aurum GP practices in England.

### Study design and population

This study employed a retrospective cohort design. Our study population was drawn from a cohort of individuals registered at linkage-eligible CPRD Aurum GP practices who had been diagnosed with IPF on or after the start of our study period (1 January 2010). In order to be eligible for inclusion in our study, patients also had to have received their IPF diagnosis before the end of our study period (31 December 2019), be male or female, aged at least 40 years at the time of their IPF diagnosis, meet CPRD-defined data quality criteria and have at least one year of continuous registration with their current GP prior to their IPF diagnosis (baseline period). For each participant, the start of follow up was defined as the latest of the following dates: date of registration with their GP practice, start of the study period (i.e., 1/1/2010), date of their 40th birthday or date of IPF diagnosis. It concluded on the earliest of: last day of practice registration, the last day of practice data collection, date of death or the end of our study (31/12/2019).

### Risk factors

Selection of risk factor variables was based on a combination of a literature review, a priori clinical knowledge and data availability. Included variables thus comprised demographic characteristics [sex, age at IPF diagnosis (40–59, 60–69, 70–79, 80+ years), socioeconomic status (IMD quintiles), region (nine), ethnicity (white, Asian, Black, mixed, other)], lifestyle characteristics [BMI (normal, underweight, overweight, obese), smoking status (current, ex, never)] and presence or otherwise of selected comorbid diseases. Data on patients’ demographic and lifestyle characteristics were obtained from primary care data; data on socioeconomic status was extracted from linked IMD records. BMI and smoking status were determined at the time of IPF diagnosis (using the closest recorded values to this date). Where GP-recorded values were missing, BMI values were calculated from height and weight data closest to the IPF diagnosis date. Comorbid chronic obstructive pulmonary disease (COPD), asthma (adult-onset), lung cancer, gastro-oesophageal reflux disease (GORD), hiatus hernia, dementia, depression/anxiety, diabetes and cardiovascular diseases (pulmonary arterial hypertension, ischaemic heart disease, heart failure) was determined on the basis of the presence at least one relevant clinical code in a patient’s record at any point prior to their IPF diagnosis.

### Outcomes

Our primary outcomes were referral for PR and referral for PC. Patients were considered to have been referred if their primary care record included coded evidence of a referral after their IPF diagnosis and within their follow-up period. Any documented referrals before IPF diagnosis were discounted. The criteria for determining a referral were deliberately broad, accepting any code that signified that an individual had been considered and/or offered a referral to PR or palliative/end-of-life support services or hospice care. In the case of PR referrals, we made no distinction between being assessed/considered for PR, offered a referral and commenced/completed PR. Similarly, for PC, the selected codes encompassed referrals to generic (i.e., non-disease specific) end-of-life care support services and were not limited to those specifically aimed at people with IPF.

All code lists used in this study are available to download from: https://github.com/NHLI-Respiratory-Epi/IPF-non-pharmacological-treatment.

### Data analysis

#### Descriptive analysis

Baseline characteristics were reported for the cohort as a whole and for “referred” and “non-referred” patients (separately for PR and PC services), using frequencies for categorical variables and medians for continuous variables. In addition, we explored geographical and temporal patterns in patient referrals, again separately for PR and PC. Temporal trends were assessed by calculating the proportion of patients with a current IPF diagnosis who were referred in that year, for each year of the study period. Thus, for 2010 we divided the number of referrals made in 2010 by the number of people in our study cohort with an IPF diagnosis in 2010. Given ours was an incident cohort, the 2010 denominator was simply the number of newly-diagnosed patients in 2010; however, for 2011 onwards our denominator comprised the number of newly-diagnosed cases in that year plus any previous years, less those who died or were lost to follow up for some other reason in the preceding year. We estimated temporal trends in both PR and PC referrals stratified by sex. In the case of PR, we compared referral proportions in those with and without a diagnosis of COPD, and in the case of PC we estimated the referral proportion in the subset of people who also had comorbid COPD or lung cancer. We also estimated the proportion of people who according to their primary care records had commenced or completed PR post an IPF diagnosis.

#### Statistical analysis

We used Cox regression models to explore the association between patient characteristics and referral to PR and PC, estimating crude and adjusted hazard ratios (HR) for each risk factor listed above. Owing to a high level of missingness in BMI (~30%), our final adjusted model excluded BMI as a covariate. However, a comparison of models with and without BMI (complete-case analysis) revealed that inclusion of BMI had little impact on the HRs for the other covariates. In other sensitivity analyses, we added a cluster option to account for variations in referral decisions between different GP practices and additionally adjusted for year of diagnosis (given evidence that the likelihood of referral increased over the time of our study). We also conducted subgroup analyses whereby we estimated HRs for PR referral separately for people with and without COPD, and HRs for PC referral separately for people with and without lung cancer. We used Schoenfeld residuals to check that our final model did not violate the proportional hazards assumption. Finally, we constructed a multinomial logistic regression model to investigate whether the timing of a referral to PC services was influenced by the presence of a co-diagnosis of either lung cancer or COPD. This analysis was restricted to those who died during the study period, and we categorised referrals which occurred within a month of death as “late” and those which occurred more than 1 year prior to death as “early”.

All analyses were performed using Stata v17 (StataCorp LLC, TX, USA).

### Ethical considerations

CPRD has NHS Health Research Authority (HRA) Research Ethics Committee (REC) approval to allow the collection and release of anonymised primary care data for observational research [NHS HRA REC reference number: 05/MRE04/87]. Each year CPRD obtains Section 251 regulatory support through the HRA Confidentiality Advisory Group (CAG), to enable patient identifiers, without accompanying clinical data, to flow from CPRD contributing GP practices in England to NHS Digital, for the purposes of data linkage [CAG reference number: 21/CAG/0008]. The protocol for this research was approved by CPRD’s Research Data Governance (RDG) Process (protocol number: 23_003015) and the approved protocol is available upon request. Linked pseudonymised data was provided for this study by CPRD. Data is linked by NHS Digital, the statutory trusted third party for linking data, using identifiable data held only by NHS Digital. Select general practices consent to this process at a practice level with individual patients having the right to opt-out.

This study is based in part on data from the Clinical Practice Research Datalink obtained under license from the UK Medicines and Healthcare products Regulatory Agency. The data is provided by patients and collected by the NHS as part of their care and support. The Office for National Statistics (ONS) was the provider of the ONS Data contained within the CPRD Data and maintains a Copyright © [2024]. Linked data were re-used with the permission of The Health & Social Care Information Centre, all rights reserved. The interpretation and conclusions contained in this study are those of the author/s alone.

### Reporting summary

Further information on research design is available in the Nature Research Reporting Summary linked to this article.

## Supplementary information

Supplementary material

Reporting Summary

## Data Availability

Data are available on request from the CPRD. Their provision requires the purchase of a license, and this license does not permit the authors to make them publicly available to all. This work used data from the version collected in February 2022. To allow identical data to be obtained by others, via the purchase of a license, the code lists will be provided upon request. Licenses are available from the CPRD (http://www.cprd.com): The Clinical Practice Research Datalink Group, The Medicines and Healthcare products Regulatory Agency, 10 South Colonnade, Canary Wharf, London E14 4PU.
