# Supplementary material for: Referral to pulmonary rehabilitation and palliative care services in people with idiopathic pulmonary fibrosis in England, 2010–2019
Source: NPJ Prim Care Respir Med. 2024 Oct 9;34:27. doi: 10.1038/s41533-024-00387-6 (PMC11464758; doi:10.1038/s41533-024-00387-6)
Supplement: Supplementary file 1 — Supplementary material [file 41533_2024_387_MOESM1_ESM.docx]

**Supplementary material**

Referral to pulmonary rehabilitation and palliative care services in people with idiopathic pulmonary fibrosis in England, 2010–2019

**Authors**: Ann D Morgan^1^, Hakeem Khan^1^, Dr Peter George^2^, Jennifer K Quint^1^

**Affiliations**:

1. Respiratory EHR Group, National Heart and Lung Institute/School of Public Health, Imperial College London, London, UK

2. Interstitial Lung Disease Unit, Royal Brompton Hospital and Harefield NHS Foundation Trust, London, UK/National Heart and Lung Institute, Imperial College London, London, UK

Corresponding author: [a.morgan15@imperial.ac.uk](mailto:a.morgan15@imperial.ac.uk)

**__________________________________________________________________________________**

**List of items**

**Figures**

**Figure S1** | Study cohort (patient flow chart)

**Figure S2** | Proportion of diagnosed patients referred to a) pulmonary rehabilitation and
b) palliative care services by sex, 2010–2019

**Tables**

**Table S1** | Referrals to pulmonary rehabilitation and palliative care services among a cohort of 17,071 people with IPF by geographical region, 2010–2019

**Table S2** | Crude and adjusted hazard ratios for the associations between selected patient characteristics and referral to pulmonary rehabilitation and palliative care services among a cohort of people diagnosed with IPF between 2010 and 2019 in England

**Table S3** | Crude and adjusted hazard ratios for the associations between selected patient characteristics and referral to pulmonary rehabilitation among a cohort of people diagnosed with IPF between 2010 and 2019 in England, with and without a prior diagnosis of COPD

**Table S4** | Crude and adjusted hazard ratios for the associations between selected patient characteristics and referral to palliative care services among a cohort of people diagnosed with IPF between 2010 and 2019 in England, with and without a prior diagnosis of lung cancer

| Patients with at least one IPF record in CPRD Aurum and flagged as “regular” and “acceptable”  N = 36,963 | |  |  |
| --- | --- | --- | --- |
|  |  |  | Excluded patients not registered at an  UTS GP practice (n= 360) |
|  |  |  |  |
| IPF patients registered at GP practices which meet  CPRD quality criteria  N = 36,603 | |  |  |
|  |  |  | Excluded patients not registered at a  linked GP practice  (n= 1,545) |
|  |  |  |  |
| IPF patients registered at a linked GP practice  N = 35,058 | |  |  |
|  |  |  | Excluded patients aged under 40 years  at time of IPF diagnosis  (n= 278) |
|  |  |  |  |
| IPF patients aged at least 40 years at the  time of their diagnosis  N = 34,780 | |  |  |
|  |  |  | Excluded patients who received their IPF diagnosis outside the study period (n= 13,323) |
|  |  |  |  |
| Patients diagnosed with IPF between 1/1/2010 and 31/12/2019 (“incident” cases) N = 21,548 | |  |  |
|  |  |  | Excluded patients with less than 12 months  of follow up prior to diagnosis  (n= 4,447) |
|  |  |  |  |
| Total number of patients eligible for inclusion  in study cohort  N = 17,071 | |  |  |

**Figure S1: Study cohort**

CPRD: Clinical Practice Research Datalink; IPF: idiopathic pulmonary fibrosis; UTS: up to standard

1. Pulmonary rehabilitation
2. Palliative care

**Figure S2:** Proportion of diagnosed patients referred to a) pulmonary rehabilitation and
b) palliative care services by sex, 2010–2019

**Table S1:** Referrals to pulmonary rehabilitation and palliative care services among a cohort of 17,071 people with IPF by geographical region, 2010–2019

|  |  | **Pulmonary rehabilitation** | | |  | | **Palliative** **care** | | | | |
| --- | --- | --- | --- | --- | --- | --- | --- | --- | --- | --- | --- |
| **Region** | **No. of cases** | **No. referred** | **Referral proportion^a^** | **95% CI** |  | **No. referred** | | **Referral proportion^a^** | **95% CI** | |  |
| North East | 735 | 104 | 14.15 | 11.63–16.67 |  | 147 | | 20.00 | 17.11–22.89 |  |  |
| North West | 4,338 | 653 | 15.05 | 13.99–16.12 |  | 879 | | 20.26 | 19.07–21.46 |  |  |
| Yorkshire/Humber | 736 | 90 | 12.23 | 9.86–14.60 |  | 130 | | 17.66 | 14.91–20.42 |  |  |
| East Midlands | 303 | 43 | 14.19 | 10.26–18.12 |  | 61 | | 20.13 | 15.62–24.65 |  |  |
| West Midlands | 2924 | 301 | 10.29 | 9.19–11.40 |  | 603 | | 20.62 | 19.16–22.09 | |  |
| East of England | 564 | 64 | 11.35 | 8.73–13.97 |  | 143 | | 25.35 | 21.76–28.95 | |  |
| London | 1,863 | 190 | 10.20 | 8.52–11.57 |  | 340 | | 18.25 | 16.50–20.00 | |  |
| South East | 3,354 | 372 | 11.09 | 10.03–12.15 |  | 621 | | 18.52 | 17.20–19.83 | |  |
| South West | 2,016 | 201 | 9.97 | 8.66–11.28 |  | 340 | | 16.87 | 15.23–18.50 | |  |
| Unknown | 238 | 24 | 10.08 | 6.29–13.91 |  | 45 | | 18.91 | 13.93–23.88 | |  |
| Total (England) | 17,071 | 2,042 | 12.0 | 11.5–12.5 |  | 3,309 | | 19.4 | 18.8–20.0 | |  |

CI, confidence interval

**^a^** Number referred as a percentage of those with an IPF diagnosis.

**Table S2:** Crude and adjusted hazard ratios for the associations between selected patient characteristics and referral to pulmonary rehabilitation and palliative care services among a cohort of people diagnosed with IPF between 2010 and 2019 in England

| **Patient**  **characteristic** | **Pulmonary rehabilitation** | | | | | |  | **Palliative care** | | | | | |
| --- | --- | --- | --- | --- | --- | --- | --- | --- | --- | --- | --- | --- | --- |
|  | **Unadjusted** | | | **Adjusted^a^** | | |  | **Unadjusted** | | | **Adjusted^a^** | | |
|  | **HR** | **95% CI** | **P-value** | **HR** | **95% CI** | **P-value** |  | **HR** | **95% CI** | **P-value** | **HR** | **95% CI** | **P-value** |
| **Sex** |  |  |  |  |  |  |  |  |  |  |  |  |  |
| Male | 1 | - | - | 1 | - | - |  | 1 | - | - | 1 | - | - |
| Female | 0.70 | 0.64-0.77 | <0.001 | 0.74 | 0.67-0.82 | **<0.001** |  | 0.81 | 0.76-0.87 | <0.001 | **0.84** | 0.78-0.90 | **<0.001** |
| **Age group (years)** | |  |  |  |  |  |  |  |  |  |  |  |  |
| 40–59 | 1 | - | - | 1 | - | - |  | 1 | - | - | 1 | - |  |
| 60–69 | 1.25 | 1.06-1.49 | 0.010 | 1.16 | 0.97-1.38 | 0.097 |  | 1.84 | 1.53-2.22 | <0.001 | **1.71** | 1.41-2.06 | **<0.001** |
| 70–79 | 1.23 | 1.05-1.45 | 0.012 | 1.18 | 0.99-1.39 | 0.059 |  | 2.49 | 2.09-2.97 | <0.001 | **2.21** | 1.85-2.65 | **<0.001** |
| 80+ | 0.86 | 0.72-1.03 | 0.101 | 0.91 | 0.75-1.09 | 0.295 |  | 3.60 | 3.02-4.30 | <0.001 | **3.15** | 2.62-3.79 | **<0.001** |
| **BMI** |  |  |  |  |  |  |  |  |  |  |  |  |  |
| Normal | 1 | - | - | - | - | - |  | 1 | - | - | - | - | - |
| Underweight | 1.24 | 0.94-1.65 | 0.132 | - | - | - |  | 1.63 | 1.35-1.99 | <0.001 | - | - | - |
| Overweight | 1.05 | 0.93-1.18 | 0.431 | - | - | - |  | 0.81 | 0.74-0.89 | <0.001 | - | - | - |
| Obese | 1.13 | 0.99-1.28 | 0.065 | - | - | - |  | 0.68 | 0.61-0.76 | <0.001 | - | - | - |
| **Smoking status** |  |  |  |  |  |  |  |  |  |  |  |  |  |
| Non-smoker | 1 | - |  | 1 | - | - |  | 1 | - | - | 1 | - | - |
| Ex-smoker | 2.14 | 1.77-2.57 | <0.001 | **1.49** | 1.23-1.80 | **<0.001** |  | 1.20 | 1.07-1.34 | 0.001 | 1.03 | 0.91-1.15 | 0.660 |
| Current smoker | 2.94 | 2.41–3.59 | <0.001 | **1.77** | 1.44-2.18 | **<0.001** |  | 1.03 | 0.90-1.18 | 0.690 | 0.99 | 0.86-1.14 | 0.850 |
| **Ethnicity** |  |  |  |  |  |  |  |  |  |  |  |  |  |
| White | 1 | - | - | 1 | - | - |  | 1 | - | - | 1 | - | - |
| Asian | 0.73 | 0.59-0.91 | 0.004 | 1.01 | 0.80-1.26 | 0.962 |  | 0.70 | 0.59-0.83 | <0.001 | **0.81** | 0.68-0.98 | **0.029** |
| Black | 0.57 | 0.35-0.94 | 0.026 | 0.71 | 0.43-1.17 | 0.179 |  | 0.39 | 0.24-0.61 | <0.001 | **0.51** | 0.32-0.82 | **0.005** |
| Mixed | 1.07 | 0.54-2.15 | 0.839 | 1.32 | 0.66-2.66 | 0.429 |  | 0.71 | 0.37-1.36 | 0.497 | 0.78 | 0.40-1.50 | 0.451 |
| Other | 0.95 | 0.54-1.67 | 0.848 | 1.13 | 0.64-2.00 | 0.679 |  | 0.52 | 0.29-0.93 | 0.250 | 0.63 | 0.34-1.13 | 0.121 |
| Unknown | 0.47 | 0.19-1.12 | 0.089 | 0.57 | 0.24-1.37 | 0.209 |  | 1.52 | 1.03-2.25 | 0.369 | 1.92 | 1.29-2.84 | 0.001 |
| **Region** |  |  |  |  |  |  |  |  |  |  |  |  |  |
| North East | 1 | - | - | 1 | - | - |  | 1 | - | - | 1 | - | - |
| North West | 1.18 | 0.96-1.45 | 0.115 | 1.15 | 0.93-1.41 | 0.191 |  | 1.13 | 0.95-1.34 | 0.175 | 1.12 | 0.94-1.34 | 0.206 |
| Yorkshire/ Humber | 0.87 | 0.66-1.15 | 0.334 | 0.87 | 0.65-1.15 | 0.331 |  | 0.90 | 0.71-1.14 | 0.368 | 0.89 | 0.70-1.13 | 0.353 |
| East Midlands | 1.12 | 0.79-1.60 | 0.526 | 1.30 | 0.91-1.86 | 0.153 |  | 1.12 | 0.83-1.51 | 0.453 | 1.13 | 0.84-1.53 | 0.411 |
| West Midlands | 0.79 | 0.63-0.98 | 0.036 | 0.93 | 0.66-1.03 | 0.095 |  | 1.14 | 0.96-1.37 | 0.143 | 1.16 | 0.97-1.39 | 0.104 |
| East of England | 0.75 | 0.55-1.03 | 0.073 | 0.79 | 0.58-1.08 | 0.143 |  | 1.25 | 1.00-1.58 | 0.054 | **1.30** | 1.03-1.64 | **0.029** |
| London | 0.69 | 0.54-0.87 | 0.002 | 0.72 | 0.56-0.92 | **0.008** |  | 0.89 | 0.73-1.08 | 0.237 | 0.98 | 0.80-1.19 | 0.833 |
| South East | 0.84 | 0.67-1.04 | 0.108 | 0.87 | 0.70-1.09 | 0.223 |  | 1.03 | 0.86-1.23 | 0.759 | 1.00 | 0.83-1.20 | 0.991 |
| South West | 0.73 | 0.58-0.92 | 0.009 | 0.75 | 0.59-0.96 | **0.020** |  | 0.90 | 0.74-1.09 | 0.267 | 0.88 | 0.72-1.06 | 0.183 |
| **IMD (quintiles)** |  |  |  |  |  |  |  |  |  |  |  |  |  |
| Least deprived | 1 | - | - | 1 | - | - |  | 1 | - | - | 1 | - | - |
| Low deprivation | 1.12 | 0.97–1.28 | 0.120 | 1.03 | 0.89-1.19 | 0.682 |  | 1.02 | 0.92-1.13 | 0.713 | 1.00 | 0.89-1.11 | 0.942 |
| Moderate deprivation | 1.10 | 0.95–1.27 | 0.194 | 1.01 | 0.87-1.17 | 0.889 |  | 1.07 | 0.96-1.19 | 0.243 | 1.08 | 0.97-1.21 | 0.170 |
| High deprivation | 1.18 | 1.02-1.36 | 0.022 | 1.04 | 0.89-1.20 | 0.626 |  | 1.06 | 0.95-1.18 | 0.313 | 1.10 | 0.98-1.23 | 0.094 |
| Most deprived | 1.41 | 1.23-1.62 | <0.001 | 1.05 | 0.91-1.21 | 0.533 |  | 1.00 | 0.90-1.11 | 0.974 | 1.02 | 0.91-1.15 | 0.693 |
| ***Comorbidities (at baseline)*** | | | | | | | | | | | | | |
| **COPD** |  |  |  |  |  |  |  |  |  |  |  |  |  |
| No | 1 | - |  | 1 | - | - |  | 1 | - | - | 1 | - | - |
| Yes | 4.51 | 4.12-4.93 | <0.001 | **4.04** | 3.68-4.45 | **<0.001** |  | 1.37 | 1.25-1.49 | <0.001 | **1.32** | 1.20-1.45 | **<0.001** |
| **Asthma** |  |  |  |  |  |  |  |  |  |  |  |  |  |
| No | 1 | - | - | 1 | - | - |  | 1 | - | - | 1 | - | - |
| Yes | 1.40 | 1.26-1.56 | <0.001 | 1.00 | 0.89-1.11 | 0.943 |  | 0.91 | 0.83-1.00 | 0.057 | 0.90 | 0.82-0.99 | 0.028 |
| **Lung cancer** |  |  |  |  |  |  |  |  |  |  |  |  |  |
| No | 1 | - | - | 1 | - | - |  | 1 | - | - | 1 | - | - |
| Yes | 2.19 | 1.45-3.30 | <0.001 | 1.38 | 0.91-2.08 | 0.131 |  | 2.96 | 2.22-3.95 | <0.001 | **2.83** | 2.11- 3.78 | **<0.001** |
| **PAH** |  |  |  |  |  |  |  |  |  |  |  |  |  |
| No | 1 | - | - | 1 | - | - |  | 1 | - | - | 1 | - | - |
| Yes | 1.30 | 0.93-1.83 | 0.127 | 1.23 | 0.87-1.74 | 0.243 |  | 1.94 | 1.54-2.43 | <0.001 | **1.64** | 1.30-2.07 | **<0.001** |
| **GORD** |  |  |  |  |  |  |  |  |  |  |  |  |  |
| No | 1 | - | - | 1 | - | - |  | 1 | - | - | 1 | - | - |
| Yes | 1.14 | 1.03-1.26 | 0.009 | 1.09 | 0.98-1.21 | 0.113 |  | 1.05 | 0.97-1.13 | 0.262 | 1.01 | 0.93-1.10 | 0.799 |
| **Hernia** |  |  |  |  |  |  |  |  |  |  |  |  |  |
| No | 1 | - | - | 1 | - | - |  | 1 | - | - | 1 | - | - |
| Yes | 1.10 | 0.97-1.24 | 0.137 | 1.04 | 0.92-1.19 | 0.518 |  | 1.18 | 1.08-1.30 | 0.001 | 1.06 | 0.96-1.17 | 0.239 |
| **Heart failure** |  |  |  |  |  |  |  |  |  |  |  |  |  |
| No | 1 | - | - | 1 | - | - |  | 1 | - | - | 1 | - | - |
| Yes | 1.07 | 0.91-1.24 | 0.417 | 1.05 | 0.89-1.23 | 0.582 |  | 1.64 | 1.48-1.81 | <0.001 | **1.30** | 1.16-1.44 | **<0.001** |
| **IHD** |  |  |  |  |  |  |  |  |  |  |  |  |  |
| No | 1 | - | - | 1 | - | - |  | 1 | - | - | 1 | - | - |
| Yes | 1.02 | 0.93-1.13 | 0.634 | **0.85** | 0.77-0.95 | **0.003** |  | 1.41 | 1.31-1.52 | <0.001 | **1.14** | 1.06-1.23 | **0.001** |
| **Diabetes** |  |  |  |  |  |  |  |  |  |  |  |  |  |
| No | 1 | - | - | 1 | - | - |  | 1 | - | - | 1 | - | - |
| Yes | 1.10 | 1.00-1.22 | 0.059 | 1.08 | 0.97-1.20 | 0.145 |  | 1.21 | 1.11-1.31 | <0.001 | **1.13** | 1.04-1.23 | **0.003** |
| **Stroke** |  |  |  |  |  |  |  |  |  |  |  |  |  |
| No | 1 | - | - | 1 | - | - |  | 1 | - | - | 1 | - | - |
| Yes | 1.13 | 0.98-1.30 | 0.098 | 1.09 | 0.94-1.26 | 0.269 |  | 1.25 | 1.12-1.39 | <0.001 | 1.06 | 0.95-1.18 | 0.297 |
| **Dementia** |  |  |  |  |  |  |  |  |  |  |  |  |  |
| No | 1 | - | - |  |  |  |  | 1 | - | - | 1 | - | - |
| Yes | 0.52 | 0.33-0.81 | 0.004 | **0.48** | 0.30-0.76 | **0.002** |  | 1.83 | 1.49-2.23 | <0.001 | **1.45** | 1.18-1.79 | **<0.001** |
| **Depression** |  |  |  |  |  |  |  |  |  |  |  |  |  |
| No | 1 | - | - |  |  |  |  | 1 | - | - | 1 | - | - |
| Yes | 1.21 | 1.11-1.33 | <0.001 | 1.12 | 1.01-1.23 | 0.034 |  | 1.07 | 0.99-1.15 | 0.098 | **1.14** | 1.05-1.24 | **0.001** |

BMI, body mass index; CI, confidence interval; COPD, chronic obstructive pulmonary disease; GORD, gastro-oesophageal reflux disease; HR, hazard ratio; IHD, ischaemic heart disease; IMD, index of multiple deprivation; PAH, pulmonary artery hypertension

^a^  Mutually adjusted for all other listed variables with the exception of body mass index.

**Table S3**: Crude and adjusted hazard ratios for the associations between selected patient characteristics and referral to pulmonary rehabilitation among a cohort of people diagnosed with IPF between 2010 and 2019 in England, with and without a prior diagnosis of COPD

| **Patient**  **characteristic** | **Without COPD (n=14,297)** | | | | | |  | **With COPD (n=2,774)** | | | | | |
| --- | --- | --- | --- | --- | --- | --- | --- | --- | --- | --- | --- | --- | --- |
|  | **Unadjusted** | | | **Adjusted^a^** | | |  | **Unadjusted** | | | **Adjusted^a^** | | |
|  | **HR** | **95% CI** | **P-value** | **HR** | **95% CI** | **P-value** |  | **HR** | **95% CI** | **P-value** | **HR** | **95% CI** | **P-value** |
| **Sex** |  |  |  |  |  |  |  |  |  |  |  |  |  |
| Male | 1 | - | - | 1 | - | - |  | 1 | - | - | 1 | - | - |
| Female | 0.69 | 0.61-0.77 | <0.001 | 0.69 | 0.61-0.78 | **<0.001** |  | 0.86 | 0.74-1.00 | 0.051 | **0.82** | 0.71-0.96 | **0.014** |
| **Age group (years)** |  |  |  |  |  |  |  |  |  |  |  |  |  |
| 40–59 | 1 | - | - | 1 | - | - |  | 1 | - | - | 1 | - |  |
| 60–69 | 1.15 | 0.93-1.41 | 0.203 | 1.16 | 0.93-1.44 | 0.182 |  | 1.14 | 0.84-1.53 | 0.402 | 1.19 | 0.88-1.61 | 0.254 |
| 70–79 | 1.12 | 0.92-1.37 | 0.256 | 1.18 | 0.95-1.45 | 0.131 |  | 1.11 | 0.84-1.47 | 0.478 | 1.25 | 0.93-1.67 | 0.144 |
| 80+ | 0.79 | 0.64-0.98 | 0.031 | 0.88 | 0.69-1.10 | 0.258 |  | 0.88 | 0.65-1.18 | 0.394 | 0.99 | 0.72-1.37 | 0.975 |
| **BMI** |  |  |  |  |  |  |  |  |  |  |  |  |  |
| Normal | 1 | - | - | - | - | - |  | 1 | - | - | - | - | - |
| Underweight | 1.21 | 0.79-1.84 | 0.380 | - | - | - |  | 1.13 | 0.77-1.67 | 0.531 | - | - | - |
| Overweight | 1.20 | 1.02-1.42 | 0.029 | - | - | - |  | 1.04 | 0.87-1.24 | 0.664 | - | - | - |
| Obese | 1.29 | 1.09-1.53 | 0.004 | - | - | - |  | 1.06 | 0.88-1.27 | 0.551 | - | - | - |
| **Smoking status** |  |  |  |  |  |  |  |  |  |  |  |  |  |
| Non-smoker | 1 | - |  | 1 | - | - |  | 1 | - | - | 1 | - | - |
| Ex-smoker | 1.57 | 1.28-1.91 | <0.001 | **1.42** | 1.16-1.74 | **0.001** |  | 2.13 | 1.14-3.98 | 0.018 | **1.87** | 1.00-3.52 | **0.051** |
| Current smoker | 2.03 | 1.62-2.54 | <0.001 | **1.84** | 1.46-2.32 | **<0.001** |  | 2.36 | 1.25-4.45 | 0.008 | **2.02** | 1.06-3.84 | **0.032** |
| **Ethnicity** |  |  |  |  |  |  |  |  |  |  |  |  |  |
| White | 1 | - | - | 1 | - | - |  | 1 | - | - | 1 | - | - |
| Asian | 0.90 | 0.70-1.15 | 0.404 | 1.05 | 0.81-1.37 | 0.719 |  | 0.74 | 0.46-1.17 | 0.198 | 0.88 | 0.54-1.43 | 0.605 |
| Black | 0.66 | 0.36-1.19 | 0.166 | 0.89 | 0.48-1.64 | 0.712 |  | 0.47 | 0.19-1.13 | 0.092 | 0.50 | 0.21-1.22 | 0.128 |
| Mixed | 1.91 | 0.34-2.44 | 0.855 | 1.08 | 0.41-2.90 | 0.871 |  | 1.40 | 0.52-3.73 | 0.506 | 1.81 | 0.67-4.90 | 0.242 |
| Other | 1.19 | 0.62-2.30 | 0.599 | 1.42 | 0.73-2.75 | 0.299 |  | 0.60 | 0.49-1.86 | 0.374 | 0.72 | 0.23-2.25 | 0.569 |
| **Region** |  |  |  |  |  |  |  |  |  |  |  |  |  |
| North East | 1 | - | - | 1 | - | - |  | 1 | - | - | 1 | - | - |
| North West | 1.44 | 1.08-1.92 | 0.013 | **1.52** | **1.14-2.03** | **0.004** |  | 0.81 | 0.60-1.09 | 0.167 | 0.79 | 0.59-1.08 | 0.138 |
| Yorkshire/ Humber | 1.12 | 0.77-1.62 | 0.545 | 1.15 | 0.79-1.66 | 0.475 |  | .0.61 | 0.39-0.95 | 0.029 | **0.60** | **0.38-0.95** | **0.028** |
| East Midlands | 1.20 | 0.74-1.94 | 0.463 | 1.33 | 0.82-2.16 | 0.252 |  | 1.31 | 0.77-2.22 | 0.319 | 1.35 | 0.79-2.31 | 0.272 |
| West Midlands | 0.89 | 0.65-1.21 | 0.453 | 0.94 | 0.69-1.28 | 0.676 |  | 0.71 | 0.51-0.98 | 0.038 | 0.71 | 0.51-0.99 | 0.044 |
| East of England | 1.02 | 0.68-1.51 | 0.930 | 1.07 | 0.71-1.60 | 0.754 |  | 0.49 | 0.29-0.83 | 0.008 | **0.50** | 0.29-0.86 | **0.012** |
| London | 0.80 | 0.58-1.11 | 0.185 | 0.85 | 0.61-1.20 | **0.357** |  | 0.55 | 0.39-0.79 | 0.001 | **0.57** | 0.39-0.82 | **0.002** |
| South East | 1.01 | 0.75-1.36 | 0.942 | 1.09 | 0.80-1.48 | 0.591 |  | 0.64 | 0.47-0.88 | 0.007 | **0.66** | 0.47-0.91 | **0.012** |
| South West | 0.86 | 0.62-1.19 | 0.362 | 0.87 | 0.63-0.21 | **0.415** |  | 0.64 | 0.45-0.91 | 0.012 | **0.63** | 0.44-0.91 | **0.013** |
| **IMD (quintiles)** |  |  |  |  |  |  |  |  |  |  |  |  |  |
| Least deprived | 1 | - | - | 1 | - | - |  | 1 | - | - | 1 | - | - |
| Low deprivation | 1.09 | 0.92–1.29 | 0.304 | 1.04 | 0.88-1.24 | 0.618 |  | 1.01 | 0.79-1.29 | 0.946 | 1.04 | 0.80-1.34 | 0.780 |
| Moderate deprivation | 1.03 | 0.86–1.23 | 0.742 | 1.04 | 0.87-1.25 | 0.656 |  | 1.00 | 0.78-1.28 | 0.995 | 1.02 | 0.79-1.32 | 0.879 |
| High deprivation | 1.00 | 0.83-1.20 | 0.995 | 0.97 | 0.80-1.17 | 0.731 |  | 1.17 | 0.92-1.48 | 0.198 | 1.21 | 0.94-1.55 | 0.134 |
| Most deprived | 1.07 | 0.90-1.28 | 0.446 | 0.96 | 0.79-1.16 | 0.659 |  | 1.24 | 0.99-1.54 | 0.060 | 1.22 | 0.97-1.55 | 0.092 |
| ***Comorbidities (at baseline)*** | | | | | | | | | | | | | |
| **Asthma** |  |  |  |  |  |  |  |  |  |  |  |  |  |
| No | 1 | - | - | 1 | - | - |  | 1 | - | - | 1 | - | - |
| Yes | 0.97 | 0.82-1.14 | 0.705 | 0.98 | 0.83-1.16 | 0.838 |  | 0.96 | 0.83-1.11 | 0.574 | 0.99 | 0.86-1.15 | 0.929 |
| **Lung cancer** |  |  |  |  |  |  |  |  |  |  |  |  |  |
| No | 1 | - | - | 1 | - | - |  | 1 | - | - | 1 | - | - |
| Yes | 0.89 | 0.34-2.39 | 0.824 | 0.82 | 0.31-2.20 | 0.699 |  | 1.60 | 1.01-2.52 | 0.044 | 1.58 | 0.99-2.52 | 0.053 |
| **PAH** |  |  |  |  |  |  |  |  |  |  |  |  |  |
| No | 1 | - | - | 1 | - | - |  | 1 | - | - | 1 | - | - |
| Yes | 1.04 | 0.63-1.74 | 0.873 | 1.19 | 0.71-1.99 | 0.604 |  | 1.30 | 0.82-2.04 | 0.264 | 1.31 | 0.81-2.10 | 0.269 |
| **GORD** |  |  |  |  |  |  |  |  |  |  |  |  |  |
| No | 1 | - | - | 1 | - | - |  | 1 | - | - | 1 | - | - |
| Yes | 1.12 | 0.99-1.28 | 0.079 | 1.12 | 0.98-1.28 | 0.102 |  | 1.07 | 0.92-1.25 | 0.368 | 1.07 | 0.90-1.27 | 0.499 |
| **Hernia** |  |  |  |  |  |  |  |  |  |  |  |  |  |
| No | 1 | - | - | 1 | - | - |  | 1 | - | - | 1 | - | - |
| Yes | 1.06 | 0.90-1.25 | 0.456 | 1.07 | 0.90-1.27 | 0.434 |  | 1.00 | 0.83-1.21 | 0.983 | 1.01 | 0.82-1.23 | 0.953 |
| **Heart failure** |  |  |  |  |  |  |  |  |  |  |  |  |  |
| No | 1 | - | - | 1 | - | - |  | 1 | - | - | 1 | - | - |
| Yes | 0.97 | 0.79-1.20 | 0.782 | 1.01 | 0.81-1.26 | 0.933 |  | 1.02 | 0.81-1.27 | 0.883 | 1.13 | 0.89-1.44 | 0.310 |
| **IHD** |  |  |  |  |  |  |  |  |  |  |  |  |  |
| No | 1 | - | - | 1 | - | - |  | 1 | - | - | 1 | - | - |
| Yes | 1.01 | 0.89-1.15 | 0.876 | **0.91** | 0.79-1.04 | **0.162** |  | 0.85 | 0.73-0.99 | 0.041 | **0.78** | 0.67-0.92 | **0.004** |
| **Diabetes** |  |  |  |  |  |  |  |  |  |  |  |  |  |
| No | 1 | - | - | 1 | - | - |  | 1 | - | - | 1 | - | - |
| Yes | 1.14 | 1.00-1.31 | 0.047 | 1.12 | 0.98-1.29 | 0.093 |  | 1.03 | 0.88-1.21 | 0.711 | 1.01 | 0.85-1.19 | 0.949 |
| **Stroke** |  |  |  |  |  |  |  |  |  |  |  |  |  |
| No | 1 | - | - | 1 | - | - |  | 1 | - | - | 1 | - | - |
| Yes | 1.00 | 0.82-1.22 | 0.979 | 1.01 | 0.83-1.24 | 0.894 |  | 1.17 | 0.95-1.45 | 0.131 | 1.16 | 0.93-1.44 | 0.180 |
| **Dementia** |  |  |  |  |  |  |  |  |  |  |  |  |  |
| No | 1 | - | - |  |  |  |  | 1 | - | - | 1 | - | - |
| Yes | 0.40 | 0.20-0.81 | 0.011 | **0.43** | 0.21-0.86 | **0.017** |  | 0.50 | 0.27-0.90 | 0.021 | **0.49** | 0.27-0.89 | **0.020** |
| **Depression** |  |  |  |  |  |  |  |  |  |  |  |  |  |
| No | 1 | - | - |  |  |  |  | 1 | - | - | 1 | - | - |
| Yes | 1.15 | 1.01-1.31 | 0.031 | **1.16** | **1.02-1.32** | **0.028** |  | 1.07 | 0.93-1.24 | 0.352 | 1.06 | 0.91-1.25 | 0.433 |

BMI, body mass index; CI, confidence interval; COPD, chronic obstructive pulmonary disease; GORD, gastro-oesophageal reflux disease; HR, hazard ratio; IHD, ischaemic heart disease; IMD, index of multiple deprivation; PAH, pulmonary artery hypertension

^a^  Mutually adjusted for all other listed variables with the exception of body mass index and COPD.

**Table S4:** Crude and adjusted hazard ratios for the associations between selected patient characteristics and referral to palliative care services among a cohort of people diagnosed with IPF between 2010 and 2019 in England, with and without a prior diagnosis of lung cancer

| **Patient**  **characteristic** | **Without lung cancer (N=16,934)** | | | | | |  | **With lung cancer (N=137)** | | | | | |
| --- | --- | --- | --- | --- | --- | --- | --- | --- | --- | --- | --- | --- | --- |
|  | **Unadjusted** | | | **Adjusted^a^** | | |  | **Unadjusted** | | | **Adjusted^a,b^** | | |
|  | **HR** | **95% CI** | **P-value** | **HR** | **95% CI** | **P-value** |  | **HR** | **95% CI** | **P-value** | **HR** | **95% CI** | **P-value** |
| **Sex** |  |  |  |  |  |  |  |  |  |  |  |  |  |
| Male | 1 | - | - | 1 | - | - |  | 1 | - | - | 1 | - | - |
| Female | 0.80 | 0.75-0.86 | <0.001 | **0.83** | 0.77-0.90 | **<0.001** |  | 1.49 | 0.84-2.65 | 0.172 | 2.20 | 0.93-5.19 | 0.071 |
| **Age group (years)** |  |  |  |  |  |  |  |  |  |  |  |  |  |
| 40–59 | 1 | - | - | 1 | - | - |  | 1 | - | - | 1 | - |  |
| 60–69 | 1.80 | 1.49-2.17 | <0.001 | **1.67** | 1.38-2.02 | **<0.001** |  | - | - | - | - | - | - |
| 70–79 | 2.47 | 2.07-2.95 | <0.001 | **2.21** | 1.85-2.65 | **<0.001** |  | - | - | - | - | - | - |
| 80+ | 3.58 | 3.00-4.27 | <0.001 | **3.13** | 2.60-3.76 | **<0.001** |  | - | - | - | - | - | - |
| **BMI** |  |  |  |  |  |  |  |  |  |  |  |  |  |
| Normal | 1 | - | - | - | - | - |  | 1 | - | - | - | - | - |
| Underweight | 1.61 | 1.32-1.96 | <0.001 | - | - | - |  | 6.86 | 1.77-26.61 | 0.005 | - | - | - |
| Overweight | 0.81 | 0.74-0.89 | <0.001 | - | - | - |  | 0.78 | 0.34-1.77 | 0.555 | - | - | - |
| Obese | 0.68 | 0.61-0.76 | <0.001 | - | - | - |  | 0.71 | 0.26-1.93 | 0.504 | - | - | - |
| **Smoking status** |  |  |  |  |  |  |  |  |  |  |  |  |  |
| Non-smoker | 1 | - |  | 1 | - | - |  | 1 | - | - | 1 | - | - |
| Ex-smoker | 1.19 | 1.06-1.33 | 0.003 | 1.02 | 0.91-1.15 | 0.714 |  | - | - | - | - | - | - |
| Current smoker | 1.02 | 0.89-1.17 | 0.769 | 0.99 | 0.86-1.14 | 0.871 |  | - | - | - | - | - | - |
| **Ethnicity** |  |  |  |  |  |  |  |  |  |  |  |  |  |
| White | 1 | - | - | 1 | - | - |  | 1 | - | - | 1 | - | - |
| Asian | 0.70 | 0.59-0.84 | <0.001 | **0.82** | 0.68-0.98 | **0.031** |  | 0.74 | 0.40-1.35 | 0.324 | 2.78 | 0.21-37.58 | 0.439 |
| Black | 0.39 | 0.25-0.62 | <0.001 | **0.51** | 0.32-0.82 | **0.005** |  | - | - | - | - | - | - |
| Mixed | 0.71 | 0.37-1.37 | 0.031 | 0.78 | 0.40-1.50 | 0.455 |  | - | - | - | - | - | - |
| Other | 0.52 | 0.29-0.94 | 0.032 | 0.63 | 0.34-1.14 | 0.124 |  | - | - | - | - | - | - |
| **Region** |  |  |  |  |  |  |  |  |  |  |  |  |  |
| North East | 1 | - | - | 1 | - | - |  | 1 | - | - | 1 | - | - |
| North West | 1.13 | 0.95-1.35 | 0.165 | 1.13 | 0.93-1.41 | 0.171 |  | 0.47 | 0.10-2.11 | 0.325 | 0.53 | 0.10-2.11 | 0.513 |
| Yorkshire/ Humber | 0.89 | 0.70-1.13 | 0.351 | 0.90 | 0.65-1.15 | 0.383 |  | 0.55 | 0.09-3.33 | 0.518 | 0.85 | 0.09-3.33 | 0.886 |
| East Midlands | 1.09 | 0.80-1.47 | 0.599 | 1.11 | 0.91-1.86 | 0.486 |  | 1.72 | 0.29-10.32 | 0.553 | 3.00 | 0.29-10.32 | 0.384 |
| West Midlands | 1.16 | 0.96-1.39 | 0.116 | 1.18 | 0.66-1.03 | 0.078 |  | 0.27 | 0.05-1.56 | 0.144 | 0.24 | 0.05-1.56 | 0.213 |
| East of England | 1.27 | 1.01-1.61 | 0.040 | **1.32** | **0.58-1.08** | **0.019** |  | - | - | - | - | - | - |
| London | 0.88 | 0.73-1.07 | 0.212 | 0.98 | 0.56-0.92 | 0.813 |  | 0.76 | 0.16-3.68 | 0.737 | 1.38 | 0.16-3.68 | 0.773 |
| South East | 1.03 | 0.86-1.23 | 0.776 | 1.00 | 0.70-1.09 | 0.975 |  | 1.16 | 0.25-5.32 | 0.847 | 1.69 | 0.25-5.32 | 0.605 |
| South West | 0.90 | 0.74-1.09 | 0.276 | 0.87 | 0.59-0.96 | 0.180 |  | 1.12 | 0.20-6.14 | 0.900 | 2.12 | 0.20-6.14 | 0.467 |
| **IMD (quintiles)** |  |  |  |  |  |  |  |  |  |  |  |  |  |
| Least deprived | 1 | - | - | 1 | - | - |  | 1 | - | - | 1 | - | - |
| Low deprivation | 1.02 | 0.92–1.14 | 0.649 | 1.00 | 0.89-1.11 | 0.929 |  | 0.70 | 0.23-2.10 | 0.525 | 1.48 | 0.38-5.85 | 0.575 |
| Moderate deprivation | 1.06 | 0.95–1.18 | 0.284 | 1.07 | 0.96-1.20 | 0.229 |  | 1.44 | 0.59-3.53 | 0.423 | 1.35 | 0.46-3.91 | 0.584 |
| High deprivation | 1.06 | 0.95-1.18 | 0.301 | 1.10 | 0.98-1.23 | 0.102 |  | 0.88 | 0.32-2.38 | 0.798 | 1.73 | 0.46-6.51 | 0.415 |
| Most deprived | 0.99 | 0.89-1.10 | 0.850 | 1.01 | 0.90-1.14 | 0.842 |  | 1.27 | 0.55-2.92 | 0.578 | 2.21 | 0.78-6.23 | 0134 |
| ***Comorbidities (at baseline)*** | | | | | | | | | | | | | |
| **COPD** |  |  |  |  |  |  |  |  |  |  |  |  |  |
| No | 1 | - |  | 1 | - | - |  | 1 | - | - | 1 | - | - |
| Yes | 1.36 | 1.25-1.49 | <0.001 | **1.33** | 1.22-1.46 | **<0.001** |  | 0.74 | 0.40-1.35 | 0.324 | 0.69 | 0.28-1.69 | 0.417 |
| **Asthma** |  |  |  |  |  |  |  |  |  |  |  |  |  |
| No | 1 | - | - | 1 | - | - |  | 1 | - | - | 1 | - | - |
| Yes | 0.91 | 0.84-1.01 | 0.072 | 0.90 | 0.82-1.00 | 0.041 |  | 0.61 | 0.26-1.45 | 0.263 | 0.68 | 0.20-2.31 | 0.534 |
| **PAH** |  |  |  |  |  |  |  |  |  |  |  |  |  |
| No | 1 | - | - | 1 | - | - |  | 1 | - | - | 1 | - | - |
| Yes | 1.95 | 1.55-2.45 | <0.001 | **1.67** | 1.32-2.11 | **<0.001** |  | 0.90 | 0.12-6.56 | 0.916 | 0.83 | 0.09-7.96 | 0.872 |
| **GORD** |  |  |  |  |  |  |  |  |  |  |  |  |  |
| No | 1 | - | - | 1 | - | - |  | 1 | - | - | 1 | - | - |
| Yes | 1.04 | 0.96-1.13 | 0.288 | 1.01 | 0.93-1.10 | 0.791 |  | 0.87 | 0.47-1.63 | 0666 | 1.24 | 0.54-2.83 | 0.607 |
| **Hernia** |  |  |  |  |  |  |  |  |  |  |  |  |  |
| No | 1 | - | - | 1 | - | - |  | 1 | - | - | 1 | - | - |
| Yes | 1.19 | 1.08-1.31 | <0.001 | 1.07 | 0.96-1.18 | 0.214 |  | 0.78 | 0.31-1.98 | 0.603 | 0.77 | 0.22-2.68 | 0.686 |
| **Heart failure** |  |  |  |  |  |  |  |  |  |  |  |  |  |
| No | 1 | - | - | 1 | - | - |  | 1 | - | - | 1 | - | - |
| Yes | 1.64 | 1.48-1.81 | <0.001 | **1.29** | 1.15-1.43 | <0.001 |  | 3.05 | 1.07-8.63 | 0.036 | **4.93** | 1.22-20.00 | **0.025** |
| **IHD** |  |  |  |  |  |  |  |  |  |  |  |  |  |
| No | 1 | - | - | 1 | - | - |  | 1 | - | - | 1 | - | - |
| Yes | 1.43 | 1.33-1.54 | <0.001 | **1.15** | 1.06-1.24 | **0.001** |  | 0.67 | 0.28-1.58 | 0.355 | 0.67 | 0.26-1.74 | 0.416 |
| **Diabetes** |  |  |  |  |  |  |  |  |  |  |  |  |  |
| No | 1 | - | - | 1 | - | - |  | 1 | - | - | 1 | - | - |
| Yes | 1.20 | 1.11-1.30 | <0.001 | **1.13** | 1.04-1.22 | **0.005** |  | 1.81 | 0.95-3.47 | 0.072 | 1.50 | 0.63-3.59 | 0.363 |
| **Stroke** |  |  |  |  |  |  |  |  |  |  |  |  |  |
| No | 1 | - | - | 1 | - | - |  | 1 | - | - | 1 | - | - |
| Yes | 1.25 | 1.12-1.39 | <0.001 | 1.06 | 0.95-1.18 | 0.325 |  | 1.20 | 0.43-3.36 | 0.733 | 1.88 | 0.58-6.11 | 0.294 |
| **Dementia** |  |  |  |  |  |  |  |  |  |  |  |  |  |
| No | 1 | - | - |  |  |  |  | 1 | - | - | 1 | - | - |
| Yes | 1.86 | 1.52-2.28 | <0.001 | **1.48** | 1.21-1.82 | **<0.001** |  | - | - | - | - | - | - |
| **Depression** |  |  |  |  |  |  |  |  |  |  |  |  |  |
| No | 1 | - | - |  |  |  |  | 1 | - | - | 1 | - | - |
| Yes | 1.07 | 0.99-1.15 | 0.112 | **1.14** | 1.05-1.24 | **0.002** |  | 1.39 | 0.73-2.64 | 0.317 | 1.08 | 0.40-2.91 | 0.001 |

BMI, body mass index; CI, confidence interval; COPD, chronic obstructive pulmonary disease; GORD, gastro-oesophageal reflux disease; HR, hazard ratio; IHD, ischaemic heart disease; IMD, index of multiple deprivation; PAH, pulmonary artery hypertension

^a^  Mutually adjusted for all other listed variables with the exception of body mass index and lung cancer.

^b^  Due to small sample size, resulting in quasi-separation, the failure of models to reach convergence and the reporting of excessively large hazard ratios, some results have been intentionally omitted.
