# Supplementary material for: Referral to pulmonary rehabilitation and palliative care services in people with idiopathic pulmonary fibrosis in England, 2010–2019
Source: NPJ Prim Care Respir Med. 2024 Oct 9;34:27. doi: 10.1038/s41533-024-00387-6 (PMC11464758; doi:10.1038/s41533-024-00387-6)
Supplement: Supplementary file 2 — Reporting Summary [file 41533_2024_387_MOESM2_ESM.pdf]

nature portfolio

Corresponding author(s): Ann D Morgan

Last updated by author(s): May 16, 2024

Reporting Summary

Nature Portfolio wishes to improve the reproducibility of the work that we publish. This form provides structure for consistency and transparency in reporting. For further information on Nature Portfolio policies, see our Editorial Policies and the Editorial Policy Checklist.

Statistics

For all statistical analyses, confirm that the following items are present in the figure legend, table legend, main text, or Methods section.

n/a	Confirmed	
☐	☒	The exact sample size (n) for each experimental group/condition, given as a discrete number and unit of measurement
☒	☐	A statement on whether measurements were taken from distinct samples or whether the same sample was measured repeatedly
☒	☐	The statistical test(s) used AND whether they are one- or two-sided<br>*Only common tests should be described solely by name; describe more complex techniques in the Methods section.*
☐	☒	A description of all covariates tested
☐	☒	A description of any assumptions or corrections, such as tests of normality and adjustment for multiple comparisons
☐	☒	A full description of the statistical parameters including central tendency (e.g. means) or other basic estimates (e.g. regression coefficient) AND variation (e.g. standard deviation) or associated estimates of uncertainty (e.g. confidence intervals)
☐	☒	For null hypothesis testing, the test statistic (e.g. F, t, r) with confidence intervals, effect sizes, degrees of freedom and P value noted<br>*Give P values as exact values whenever suitable.*
☒	☐	For Bayesian analysis, information on the choice of priors and Markov chain Monte Carlo settings
☒	☐	For hierarchical and complex designs, identification of the appropriate level for tests and full reporting of outcomes
☐	☒	Estimates of effect sizes (e.g. Cohen's d, Pearson's r), indicating how they were calculated

Our web collection on statistics for biologists contains articles on many of the points above.

Software and code

Policy information about availability of computer code

Data collection	Patient data are provided by UK GP practices to a third party, anonymised and processed by NHS Digital services using proprietary code before being shared with researchers.
Data analysis	All code lists that were used in this piece of analysis (to identify patients with IPF and to define their demographic and clinical characteristics from their primary care records will be deposited in the public domain (on Github). Stata version 17 was used to perform all data management and processing tasks involved in the conduct of this study.

For manuscripts utilizing custom algorithms or software that are central to the research but not yet described in published literature, software must be made available to editors and reviewers. We strongly encourage code deposition in a community repository (e.g. GitHub). See the Nature Portfolio guidelines for submitting code & software for further information.

Data

Policy information about availability of data

All manuscripts must include a data availability statement. This statement should provide the following information, where applicable:

- Accession codes, unique identifiers, or web links for publicly available datasets
- A description of any restrictions on data availability
- For clinical datasets or third party data, please ensure that the statement adheres to our policy

Patient-level data used in this study while pseudo-anonymised, are only available to researchers under license and are not available in the public domain. Our manuscript includes a data availability statement to this effect.

Research involving human participants, their data, or biological material

Policy information about studies with human participants or human data. See also policy information about sex, gender (identity/presentation), and sexual orientation and race, ethnicity and racism.

Reporting on sex and gender	our analyses have been disaggregated by sex.
Reporting on race, ethnicity, or other socially relevant groupings	As we have access to patient reported data on race/ethnicity we were able to include this dimension in our analyses. UK electronic health care records are routinely linked to the UK's office of National statistics IMD data (index of multiple deprivation) which allows us to also adjust for SES in our analyses. The IMD is a widely used measure of social deprivation, comprised of 5 domains (education, income level etc), available at the patient level and expressed in the form of quintiles. These detials are mentioned in our methods section.
Population characteristics	our study population was drawn from people who are registered at a GP practice that has agreed to supply its data to CPRD for research purposes. we used our own code set to identuf people who had a diagnosis of IPF (see above).
Recruitment	We did not recruit patients to our study. See above.
Ethics oversight	CPRD patient data are only available for use to researchers with a user license and all studies which use CPRD data are subject to approval by CPRD's Research Data Governance (RDG) Process

Note that full information on the approval of the study protocol must also be provided in the manuscript.

Field-specific reporting

Please select the one below that is the best fit for your research. If you are not sure, read the appropriate sections before making your selection.

☒ Life sciences ☐ Behavioural & social sciences ☐ Ecological, evolutionary & environmental sciences

For a reference copy of the document with all sections, see nature.com/documents/nr-reporting-summary-flat.pdf

Life sciences study design

All studies must disclose on these points even when the disclosure is negative.

Sample size	At the design stage of our research, and as part of our study protocol which we submitted to CPRD for approval, we performed sample size calculations to confirm that our study population would be large enough to ensure that our planned primary analyses would be sufficiently powered.
Data exclusions	We excluded some patients from our study on the basis of pre-established criteria. these are detailed in our methods section
Replication	This is an observational as opposed to an experimental study
Randomization	See above
Blinding	Blinding is not relevvnt this study - see above

Reporting for specific materials, systems and methods

We require information from authors about some types of materials, experimental systems and methods used in many studies. Here, indicate whether each material, system or method listed is relevant to your study. If you are not sure if a list item applies to your research, read the appropriate section before selecting a response.

Materials & experimental systems

n/a	Involved in the study
☒	☐ Antibodies
☒	☐ Eukaryotic cell lines
☒	☐ Palaeontology and archaeology
☒	☐ Animals and other organisms
☒	☐ Clinical data
☒	☐ Dual use research of concern
☒	☐ Plants

Methods

n/a	Involved in the study
☒	☐ ChIP-seq
☒	☐ Flow cytometry
☒	☐ MRI-based neuroimaging

Plants

Seed stocks	*Report on the source of all seed stocks or other plant material used. If applicable, state the seed stock centre and catalogue number. If plant specimens were collected from the field, describe the collection location, date and sampling procedures.*
Novel plant genotypes	*Describe the methods by which all novel plant genotypes were produced. This includes those generated by transgenic approaches, gene editing, chemical/radiation-based mutagenesis and hybridization. For transgenic lines, describe the transformation method, the number of independent lines analyzed and the generation upon which experiments were performed. For gene-edited lines, describe the editor used, the endogenous sequence targeted for editing, the targeting guide RNA sequence (if applicable) and how the editor was applied.*
Authentication	*Describe any authentication procedures for each seed stock used or novel genotype generated. Describe any experiments used to assess the effect of a mutation and, where applicable, how potential secondary effects (e.g. second site T-DNA insertions, mosiacism, off-target gene editing) were examined.*
